# Natural melatonin fluctuation and its minimally invasive simulation in the zebra finch

**DOI:** 10.7717/peerj.1939

**Published:** 2016-04-21

**Authors:** Susanne Seltmann, Lisa Trost, Andries Ter Maat, Manfred Gahr

**Affiliations:** Department of Behavioural Neurobiology, Max Planck Institute for Ornithology, Seewiesen, Germany

**Keywords:** Melatonin, Circadian rhythm, Chronobiology, Zebra finch, Bird song

## Abstract

Melatonin is a key hormone in the regulation of circadian rhythms of vertebrates, including songbirds. Understanding diurnal melatonin fluctuations and being able to reverse or simulate natural melatonin levels are critical to investigating the influence of melatonin on various behaviors such as singing in birds. Here we give a detailed overview of natural fluctuations in plasma melatonin concentration throughout the night in the zebra finch. As shown in previous studies, we confirm that “lights off” initiates melatonin production at night in a natural situation. Notably, we find that melatonin levels return to daytime levels as early as two hours prior to the end of the dark-phase in some individuals and 30 min before “lights on” in all animals, suggesting that the presence of light in the morning is not essential for cessation of melatonin production in zebra finches. Thus, the duration of melatonin production seems not to be specified by the length of night and might therefore be less likely to directly couple circadian and annual rhythms. Additionally, we show that natural melatonin levels can be successfully simulated through a combination of light-treatment (daytime levels during subjective night) and the application of melatonin containing skin-cream (nighttime levels during subjective day). Moreover, natural levels and their fluctuation in the transition from day to night can be imitated, enabling the decoupling of the effects of melatonin, for example on neuronal activity, from sleep and circadian rhythmicity. Taken together, our high-resolution profile of natural melatonin levels and manipulation techniques open up new possibilities to answer various melatonin related questions in songbirds.

## Introduction

The “night-hormone” melatonin plays an essential role in maintaining and synchronizing the circadian rhythmicity of various physiological processes such as sleep, locomotion, temperature regulation and singing behavior of birds (Bentley, 2001; Brandstatter, 2003; Cassone & Menaker, 1984; Gwinner, Hau & Heigl, 1997). In songbirds such as the zebra finch, melatonin directly affects brain areas involved in learning and producing birdsong (Bentley & Ball, 2000; Bentley, Van’t Hof & Ball, 1999; Deregnaucourt, Saar & Gahr, 2012; Fusani & Gahr, 2015; Gahr & Kosar, 1996; Jansen et al., 2005; Whitfield-Rucker & Cassone, 1996). Mel 1B receptors, which bind melatonin, are expressed in brain nuclei HVC (formerly known as “high vocal center,” now used as a formal name; located in nidopallium) and RA (Nucleus robustus arcopallii) of the avian song control system (Fusani & Gahr, 2015), a model system for understanding motor learning in general, including language learning in humans. Both HVC and RA are involved in song learning as well as song production (Nottebohm, Stokes & Leonard, 1976; Vu, Mazurek & Kuo, 1994). To investigate the role of melatonin in these and many other processes, detailed knowledge about the circadian melatonin rhythm is necessary. Likewise, a minimally invasive method that manipulates melatonin availability is required, so that its effects can be disentangled from other covariates such as sleep.

Melatonin diel rhythmicity, with high blood plasma concentration during the night and low levels during the day, is driven by a circadian clock and fine-tuned by light (Klein, 2006; Ralph, Hedlund & Murphy, 1967; Tan et al., 2010). Melatonin synthesis and release seem to precisely follow the photoperiod and therefore couple circadian and seasonal rhythms (Perreau-Lenz et al., 2004; Ralph, Hedlund & Murphy, 1967). To date, the highest temporal resolution of published melatonin measurements in songbirds is 2h–6h (Brandstatter et al., 2000; Brandstatter et al., 2001; Janik, Dittami & Gwinner, 1992; Van’t Hof & Gwinner, 1996), which is, in part due to time points sampled, insufficient to obtain an accurate association with the timing of dawn or the onset of night. More precise measurements in non-passerine birds such as Japanese Quails (Kumar & Follett, 1993) still lack the resolution or—as well as in the mentioned songbird studies—a statistically validated tracking of changes in melatonin blood concentration. Thus, the first aim of this study is to precisely monitor and statistically validate circadian changes in melatonin levels in zebra finches with a focus on the transition between day and night.

The second aim of this study is to validate a combination of minimally invasive methods to successfully simulate natural melatonin levels as well as the circadian fluctuation of these levels. Conventional methods for increasing melatonin levels like direct hormone injections or implants are rather invasive and can therefore influence behavior. Moreover, these methods cause abnormally high peaks of hormone blood concentration after treatment and are therefore unsuitable for imitation of the natural melatonin production. Additionally, implants cause a continuous release of hormones and therefore exclude the simulation of the natural production with its circadian rhythmicity (Goymann, Trappschuh & Fusani, 2008). Similarly, while oral administration of melatonin leads to a sufficiently elevated melatonin level to change behavior (Binkley & Mosher, 1985; Heigl & Gwinner, 1994; Neddegaard & Kennaway, 1999; Pohl, 1996), neither the exact amount of melatonin taken in by the animal nor the duration of the elevation can be controlled reliably. Recent advances make the reliable and non-invasive simulation of natural melatonin levels feasible. For simulating natural melatonin levels in birds, Goymann and colleagues (2008) introduced a minimally invasive method to raise melatonin levels by applying melatonin dissolved in Eucerinum anhydricum (Beiersdorf; termed “melatonin cream” in the present paper) to the birds’ skin. This method was originally developed for wild birds to ensure the least invasive treatment possible to increase natural melatonin levels at night, but it seems to be similarly ideal for manipulating hormone levels of birds in experimental setups under various situations in the laboratory.

In addition to reliably raising melatonin levels to a certain level, the ability to suppress the natural melatonin production is equally crucial for answering various questions. To date there are no well-characterized antagonists for avian melatonin receptors available. Pinealectomy has thus far been the method of choice for suppressing natural melatonin production (Binkley, 1976; McMillan, 1972; Van’t Hof & Gwinner, 1996). However, the extremely invasive nature of this procedure may have adverse effects on behavior and does certainly impair follow-up experiments as electrophysiological measurements of brain activity. Although keeping animals in constant light might affect other bodily functions as well as behavior, it is a powerful tool to temporary suppress melatonin production (Turek, McMillan & Menaker, 1976). Consequently, establishing a combination of minimally invasive methods that allow for successful emulation of natural melatonin levels in zebra finches under laboratory conditions could provide a powerful tool for answering various questions in songbirds.

## Materials and Methods

### Animals

Adult male and female zebra finches from the breeding colony in Seewiesen, Germany were used for the experiment. Zebra finches were reared and subsequently housed socially in mixed-sex colonies in a 14/10 light/dark (LD) light cycle. For the experiment, birds were transferred and kept as couples or in mixed sex groups of five in a 14/10 (1,200 lux/<0.0001 lux) LD cycle in sound- and light-proof boxes two weeks prior to, and during, the experiment. This housing situation allowed us to minimize any outside influences as well as disturbances of birds kept in other boxes caused by the experiment itself such as melatonin treatment, catching individuals, or using night light for blood sampling at different time points during the experiment. Resampling was furthermore excluded by using individual ring numbers for each bird. All housing conditions were in accordance with EU regulations, and experimental procedures were performed in compliance with national legislation on animal experimentation (animal testing license of the government of Upper Bavaria 55.2-1-54-2531-108-10).

### Melatonin application

Two dilutions of melatonin cream, 13 µg/ml (“low concentration”) and 130 µg/ml (“high concentration”, comparable to the lowest concentration used by Goymann et al. for a different songbirds species in their publication) were prepared according to Goymann, Trappschuh & Fusani (2008) by dissolving melatonin (Sigma M 5250) in 200 µl ethanol and mixing the solution with Eucerin cream (Eucerinum anhydricum; Beiersdorf AG, Hamburg, Germany). For each treatment 50 µl cream (corresponding to 0.65 µg melatonin in the low concentration cream and 6.5 µg melatonin in the high concentration cream) was applied to the skin between the dorsal feather ridge, wing and neck of the birds. The birds were released back into the cage after the cream melted completely on the skin.

### Blood sampling

150–200 µl of blood were collected from the alar vein into heparinized micro-capillaries and centrifuged immediately after collection for ten minutes at 2500 RPM (Sigma 1–14 centrifuge). Plasma was stored at −80 °C until analysis.

### Melatonin levels in different conditions

To characterize the difference in natural melatonin levels during day and night as well as the effects of our treatments on these levels, we repeatedly took blood samples of 19 zebra finches (11 male and 8 female). Every individual was sampled once in each of the five conditions. A two-week interval was maintained between subsequent sampling events to give the birds enough time to recover. All blood samples were analyzed in a single radioimmunoassay (for details see Table S1).

First, to estimate the average melatonin level during different phases of the diurnal cycle, we took blood samples five hours after lights on as well as three hours after lights off during a normal 14/10 LD light cycle. For the night sampling, individual birds were removed from their lightproof boxes and prepared for blood collection in the dark. After reclosing the lightproof box, a night-light was turned on immediately prior to bleeding, thereby minimizing the disturbance and light exposure of all individuals including the ones bled at a single time point.

Subsequently, to evaluate the effects of constant light (LL) and melatonin treatment, we took samples of the same individuals during LL without melatonin treatment as well as during LL after treatment with the two different dilutions of melatonin cream described before. Birds were shifted to LL three days prior to sampling for each sampling event. Cream was applied at subjective “lights-off time” of the previous 14/10 LD cycle. Blood sampling took place three hours after subjective “lights-off time” or after melatonin treatment respectively. For sampling in LL, birds were caught without turning off the cage lights.

### Natural nighttime profile and influence of light/melatonin treatment

To obtain high-resolution data, we collected three series of samples over a 12-hour (normal night, no treatment)/24-hour (LL/high concentration cream treatment) / 84-hour (LL/low concentration cream treatment) period to compare natural melatonin fluctuations over night with melatonin levels observed after treatment with both melatonin cream concentrations.

A total of 277 samples were collected from different adult male and female zebra finches and analyzed in four different radioimmunoassays (for a detailed overview of sampling see Table S1). In total, seven samples had to be excluded (contaminated during sampling or lost during analysis in the lab). The extension of the sampling period for the low and the high concentration cream from 12 to 24 and 84 h respectively was a result of evaluating the samples collected in a first run, up to 12 h after treatment with melatonin.

For the natural nighttime profile a total of 96 adult birds (48 male, 47 female) were sampled (once each), covering 12 h, including the 10-hour dark period of a 14/10 LD cycle. Two series of samples were collected from birds treated with the two different melatonin cream concentrations covering a 24-hour (high concentration treatment) and an 84-hour period (low concentration treatment) respectively. For these two series, birds were shifted to LL three days prior to treatment, and 50 µl of either high or low concentration melatonin cream was applied at subjective lights-off time. For the high concentration melatonin cream series we collected blood samples from 81 adult zebra finches (39 male and 42 female) in total. For the low concentration melatonin cream series we sampled a total of 100 adult zebra finches (46 male and 54 female).

Tarsus length and body mass were measured after blood sampling in a representative group of birds treated with the low concentration cream (all birds sampled in the first 24 h after treatment) to check for size effects.

### Radioimmunoassay

The concentration of melatonin in the blood plasma of all collected samples was determined by direct radioimmunoassay (RIA) following Fusani & Gwinner (2004), Goymann et al. (2007) and Goymann, Trappschuh & Fusani (2008). The lower detection limit of the assays ranged from 4.3 to 15.6 pg/ml depending on the sampled volume and the recovery value, the upper detection limit was between 5,846 and 8,276 pg/ml (see Table S1). Intra-assay coefficient of variation (CV) ranged from 0.9 to 9%, and intra-extraction CV between 0.4 and 5.8%. The inter-assay CV was 10.4% for the four assays used for the natural nighttime profile and the influence of light/melatonin treatment was 10.4% (for more details see Table S1).

**Figure 1:**
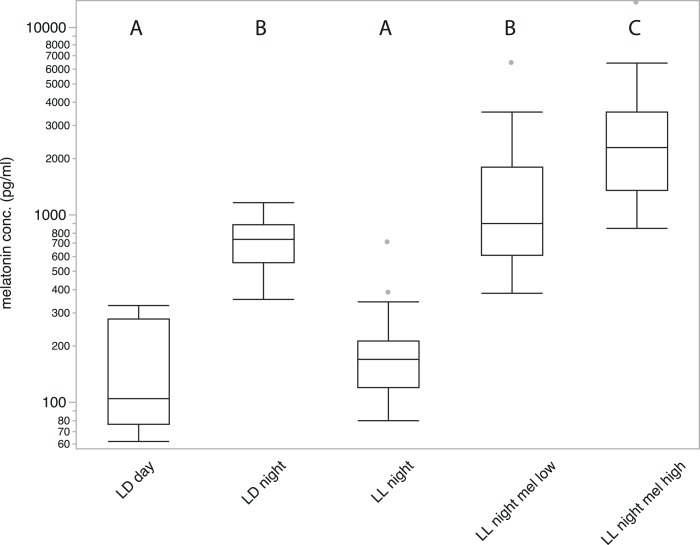
Average plasma melatonin concentrations under different conditions. Plasma melatonin concentrations of 19 adult zebra finches measured during day (LD day), night (LD night), nighttime in constant light (LL night), with high concentration melatonin cream (LL night mel high) and with low concentration melatonin cream (LL night mel low); LD indicates a normal light cycle; LL indicates constant light. Groups with different letters (A/B/C) are significantly different (Tukey’s post-hoc test, *p* < 0.01).

### Statistics

Statistical analysis was done with JMP 10.0 (SAS Institute Inc.). To test for differences in the five treatment groups (Melatonin levels in different conditions) we used a REML (restricted maximum likelihood) analysis with treatment and sex as factors, and animal ID as a random factor. Melatonin levels were log_10_-transformed to meet requirements for parametrical statistical testing. Significance of specific differences was determined posthoc using a Tukey LSD test at alpha 0.01 to minimize the chance of false positives. Raw data were also analyzed using Friedman randomized blocks followed by post-hoc testing (Fig. 1). This yielded the same result as the parametric test after log-transformation. Fluctuations of blood plasma melatonin concentrations under normal LD conditions, and after treatment with both cream concentrations under LL conditions were compared using a REML with animal ID as a random factor. A full model including sex as factor and tarsus length and weight as covariates neither showed an effect of sex or interactions with it, nor did it show any correlation of tarsus length or weight with melatonin levels. These factors were therefore excluded from further analysis. Significance of specific differences was again determined posthoc using a Tukey LSD test at alpha 0.01. Additionally, to establish when plasma melatonin levels return to daytime levels after treatment, melatonin levels at all time points after treatment with the low concentration cream were compared to the control (0.5 h before treatment) using a Dunnett’s test (Fig. S2).

## Results

### Mean melatonin levels under different conditions

Samples of all 19 birds measured in five different conditions showed substantial variation in melatonin concentration (REML fixed effects test of treatment: numerator F_4,88_ = 62.27; *P* < 0.0001, followed by Tukey’s test at *P* < 0.01; Fig. 1). Melatonin concentrations after five hours of daylight during a normal day (LD 14/10) were significantly lower than three hours after onset of night (152.75 ± 22.398 pg/ml and 716.68 ± 52.793 pg/ml respectively; *P* < 0.01, Tukey’s test). After keeping birds in LL for three days, samples collected three hours after the subjective onset of night showed melatonin concentrations comparable to those measured during daytime (203.4 ± 33.964 pg/ml; *P* < 0.01, Tukey’s test). Treated with high concentration melatonin cream and measured three hours after treatment, birds showed strongly elevated melatonin levels (3104.12 ± 782.122 pg/ml; *P* < 0.01, Tukey’s test), significantly higher than under normal nighttime conditions. Birds treated with the low concentration melatonin cream showed an average melatonin level (1539.06 ± 349 pg/ml) that was not significantly different from birds sampled under natural night conditions (*P* > 0.01, Tukey’s test). A significant difference between male and female birds could not be measured under any condition.

### Natural nighttime profile of plasma melatonin concentrations

**Figure 2:**
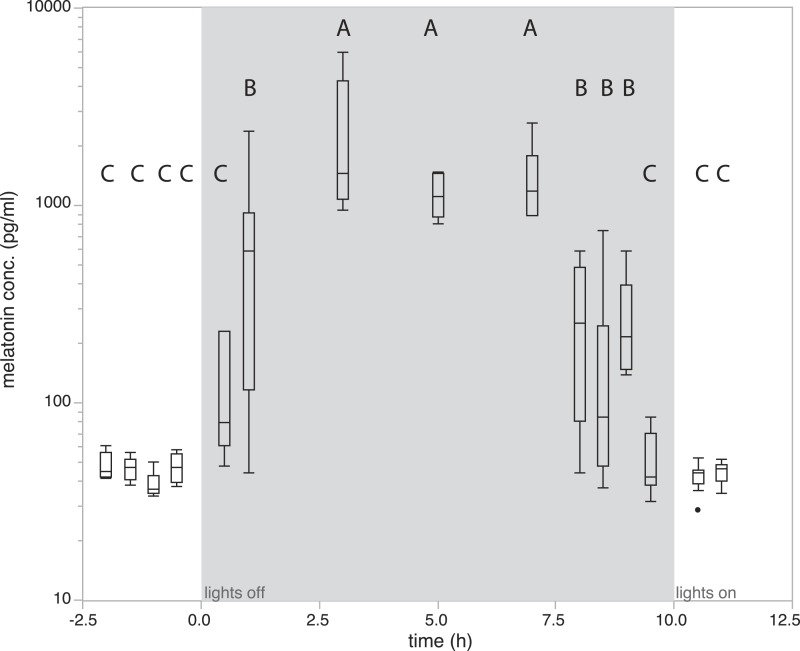
Changes in natural plasma melatonin concentration. Melatonin plasma concentrations over time in normal LD conditions. Onset of night is indicated by “lights off,” end of night by “lights on.” Melatonin concentrations under normal nighttime conditions decreased as early as two hours prior to lights on. Groups with different letters are significantly different (Tukey’s post-hoc test, *p* < 0.01).

To quantify how melatonin levels vary during a normal day/night cycle, we measured melatonin levels at 15 time points. Analysis of variance showed that levels differed significantly between sampling time points (F_14,81_ = 24.21, *P* < 0.0001). Birds kept under natural conditions showed daytime melatonin levels in all measurements before lights off. Melatonin levels were already slightly increased in the first measurement 0.5 h after lights off, a significant increase as compared to daytime levels can be observed from 1h after lights-off (685.62 ± 221.498 pg/ml; *P* > 0.01, Tukey’s post-hoc test; Fig. 2). Levels remained elevated for 7 h with small, non-significant fluctuations. Interestingly, a significant drop of melatonin levels compared to high nighttime levels already occurred as early as 2 h before lights-on in the morning (Fig. 2). Thirty minutes before lights-on, melatonin levels further decreased and reached average daytime levels (Fig. 2).

### Effects of melatonin treatment over time

**Figure 3:**
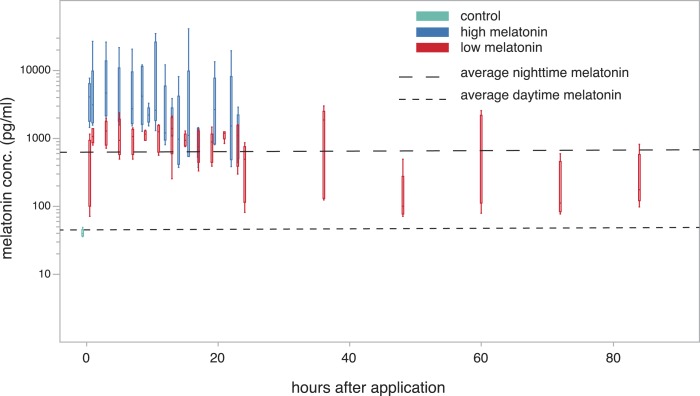
plasma melatonin concentrations after treatment with melatonin cream in constant light. Plasma melatonin concentrations under constant light conditions and after treatment with two different concentrations of melatonin cream (high: blue; low: red; control before treatment: light blue). Average daytime and nighttime levels of untreated birds (derived from Fig. 1) indicated by dashed lines. Levels in the high concentration group exceed normal nighttime levels (Tukey’s post-hoc test, *p* < 0.01). Levels in the low concentration group were not significantly different to normal nighttime levels up to 24 h after treatment. After 24 h the levels of the low concentration group were variable (see Fig. S2).

Melatonin levels showed a significant increase one hour after treatment compared to the control group (low concentration: 995.41 ± 103.216 pg/ml; high concentration: 6843.95 ± 4541.146 pg/ml; Dunnett’s test; *P* < 0.01 for both concentrations) and remained elevated for 23 h after treatment with both cream concentrations (Fig. 3). The animals treated with the high concentration even showed levels that were significantly higher than normal nighttime levels (derived from Fig. 1) during both the first and the second 10 h after treatment (99% confidence interval not encompassing average nighttime level of 716.68 pg/ml; Dunnett’s test *P* < 0.01). During these periods the normal nighttime level was within the 95% confidence interval of the low concentration animals. Twenty-three hours after treatment with the low concentration cream the first individuals show a drop to daytime melatonin levels. However, this drop was not consistent across all individuals, and in some cases concentrations remain elevated for up to 84 h (last sampled time point). There was no significant difference in the hormone levels of male and female birds and neither tarsus length nor weight of the tested birds were correlated with observed melatonin levels.

## Discussion

In this study we provide the highest temporal resolution profile of natural diel melatonin rhythmicity in zebra finches published so far, as well as a suitable combination of two minimally invasive methods including a melatonin treatment optimized for the zebra finch to manipulate and simulate melatonin levels observed under natural conditions. By collecting data with a higher temporal resolution than previous studies (Brandstatter et al., 2000; Brandstatter et al., 2001; Janik, Dittami & Gwinner, 1992; Van’t Hof & Gwinner, 1996) as well as focussing on the transition between day and night, we provide a more accurate description of the natural progression of melatonin plasma levels throughout the night in songbirds.

As expected, due to the light-sensitivity of the melatonin synthesis pathway (Klein, 2006; Klein, 2007) melatonin levels increase soon after the onset of night (lights-off) and remain high for several hours without significant fluctuation during the night. The rate-limiting step of melatonin production is the amount and activity of the enzyme AANAT (arylalkylamine N-acetyltransferase), which is essential for the synthesis of melatonin from serotonin (Klein, 2006; Klein, 2007). AANAT activity requires cAMP (cyclic adenosine monophosphate)-dependent phosphorylation and binding to 14-3-3 proteins. Light decreases cAMP levels leading to the dissociation of the AANAT /14-3-3 complex, which ultimately is followed by the proteasomal degradation of the AANAT protein (Klein, 2007; Rosiak, Michael Iuvone & Zawilska, 2005). Next to this regulation of AANAT activity, in the chicken pineal gland, melatonin production is additionally regulated by the circadian transcription of the *aanat* gene (Bernard et al., 1997; Chong, Bernard & Klein, 2000; Toller et al., 2006). Whether the fast increase of circulating melatonin levels within 30 min after lights-off observed in zebra finches reflects the dark-dependent increase in AANAT activity or is coupled to the circadian expression of the *aanat* gene remains to be seen. In some mammals at least, melatonin production can indeed be triggered by spontaneous lights-off at any given time and therefore is not systemically coupled to the onset of the night (Lu et al., 2010).

Melatonin levels show a significant drop compared to the average nighttime levels as early as two hours before lights on in a 14/10 LD rhythm. In single individuals, levels already completely returned to daytime average at this point. Thirty minutes before lights on, a drop back to daytime levels can be observed in all sampled individuals. Therefore, there is not only a mere trend to lower levels in the early morning as already observed in former publications (Brandstatter et al., 2001; Ralph, Hedlund & Murphy, 1967; Silverin et al., 2009) but a significant drop to daytime levels can be reported before lights on in the morning. These results illustrate that the absence of light is necessary but not sufficient for melatonin production. Plasma melatonin levels after treatment with melatonin cream in constant light stayed elevated consistently, also after the subjective onset of day. This could contradict a process of active late night clearance of melatonin linked to circadian rhythmicity of the animals as explanation for the nightly daytime levels observed in this study. It also indicates that the control of melatonin synthesis in the zebra finch requires light-independent mechanisms that regulate the decrease in AANAT abundance or activity before the photic turnoff mechanism becomes active as suggested already in rats (Clokie et al., 2012). Previous studies of the Japanese quail suggested that the cessation of melatonin secretion is not linked to onset of dawn and that the duration of secretion actually seems to have a certain limit (Kumar & Follett, 1993).

Since our birds were maintained in a defined environment we can exclude external photic and other environmental cues other than timing of “lights off” in the evening for triggering the pre-dawn decrease in melatonin production. Furthermore, we can exclude changes in nightly activity of the animals as an internal mechanism to trigger the decrease in melatonin since zebra finches are generally immobile in complete darkness (Wang et al., 2012).

However, the sensitivity of the bird pineal to various internal circadian and non-circadian signals is not well understood. In rats for example, insulin signaling seems to affect the pineal melatonin production (Peliciari-Garcia et al., 2010). To verify whether this early pre-dawn down-regulation of melatonin synthesis is a general pattern in birds, if, and how exactly it is influenced by seasonality, further detailed measurements of the nightly melatonin profiles in other avian species, especially in seasonal breeders, are required. Previous studies in songbirds (starlings, house sparrows, willow warblers) already show a similar trend of decreased melatonin production during the late night phase (Brandstatter et al., 2001; Ralph, Hedlund & Murphy, 1967; Silverin et al., 2009) but lacked the temporal resolution necessary to accurately pinpoint the onset of the drop and just describe low melatonin levels in general around lights on in the morning.

If similar patterns hold for songbirds in general, the observed patterns may have several interesting implications. A pre-dawn drop in melatonin production might affect important aspects of the neural control of singing as well as many other brain or peripheral functions, given the widespread expression of melatonin receptor types in the avian brain and periphery (Fusani & Gahr, 2015; Jones, Helfer & Brandstatter, 2012; Rivkees et al., 1989). This might also be interesting in the context of pre-dawn singing, as it has been suggested that the onset of singing activity is linked to the absence of melatonin (Dominoni et al., 2014; Dominoni et al., 2013), as well as influenced by light pollution (Da Silva, Valcu & Kempenaers, 2015). Our observations consequently suggest a more detailed analysis of melatonin rhythms also in other birds as well as a review of the regulation of melatonin production in songbirds in general.

The detailed insight into the natural melatonin rhythm allowed us to adjust available methods for regulating melatonin in songbirds for the zebra finch. The “low concentration cream” provides an effective way to not only simulate natural nighttime melatonin levels at any given time, but furthermore allows to reliably simulate the transition from day to night regarding melatonin fluctuations. Nevertheless, the long lasting effect of the cream currently only allows for the simulation of one such transition from day- to night-time. Melatonin levels do decline significantly 23 h after treatment with the low concentration cream in most individuals, but a general decline over all sampled animals cannot be statistically validated. Reducing the applied amount of melatonin cream could result in an earlier drop of melatonin levels in accordance to our observations under natural conditions. However, our method, in combination with the precise knowledge of circadian melatonin fluctuations, provides a powerful technique to temporarily simulate natural melatonin levels and their fluctuation in zebra finches without the confounding factors of night or sleep. Song structure for example is not thought to be influenced by stress caused by constant light. Since song learning and song production seem to be strongly related to the occurrence of melatonin (Deregnaucourt, Saar & Gahr, 2012), we expect this method to be especially suitable for studying the influence of melatonin on different aspects of birdsong at the behavioral and the neuronal level in particular.

## Supplemental Information

10.7717/peerj.1939/supp-1 Figure S1 Changes in individual plasma melatonin concentrations Individual melatonin concentrations of all birds sampled are indicated by black dots. A simple model was fitted to the time course of melatonin concentration with three levels of melatonin; mel1 as resting melatonin level, mel2 during the night, and mel3 after the drop in concentration. Two time points were estimated, t1 and t2, at the rise in melatonin level and its decrease, respectively. Best fit was obtained for mel1 = 1.72 (log pg/ml), mel2 = 2.81 (log pg/ml), mel3 = 1.83 (log pg/ml); t1 ≤ 0.5 h, t2 ≤ 8 h.

10.7717/peerj.1939/supp-2 Figure S2 Differences in plasma melatonin concentration between treatment and control Results of a Dunnett’s test comparing all sampled time points of the low concentration treatment with the control (taken before treatment, 1.59 log pg/ml). Groups marked red differ significantly from the control group (*p* < 0.05).

10.7717/peerj.1939/supp-3 Table S1 Raw data, sample overview and assay information This data set includes the individual melatonin concentration for each sample taken (Data Fig. 1 & Data Figs. 2 and 3), an overview of sampling time points as well as additional information about the different radioimmunoassays (overview samples).

## Additional Information and Declarations

### Data Availability

The following information was supplied regarding data availability: The raw data has been supplied as a Supplemental Dataset.
